# Effect of Immiscible Secondary Fluid on Particle Dynamics and Coffee Ring Characteristics during Suspension Drying

**DOI:** 10.3390/ma13153438

**Published:** 2020-08-04

**Authors:** Kevin Injoe Jung, Baek Sung Park, Seong Jae Lee, Seung Man Noh, Hyun Wook Jung

**Affiliations:** 1 Department of Chemical and Biological Engineering, Korea University, Seoul 02841, Korea; ij@grtrkr.korea.ac.kr (K.I.J.); bspark@grtrkr.korea.ac.kr (B.S.P.); 2 Department of Polymer Engineering, The University of Suwon, Hwaseong, Gyeonggi 18323, Korea; sjlee@suwon.ac.kr; 3 Research Center for Green Fine Chemicals, Korea Research Institute of Chemical Technology, Ulsan 44412, Korea

**Keywords:** autocorrelation function, coffee ring, decalin, light scattering techniques, polystyrene particles, suspension drying

## Abstract

Particle motion and coffee ring patterns in water-borne suspensions of polystyrene (PS) particle added with small amounts of secondary hydrophobic decalin are investigated during the drying of the suspension droplets, mainly employing light scattering methods. Very tiny secondary fluid insertions via high-speed agitation effectively link the particles through hydrophobic dissolution leading to the formation of multimodal particulate clusters, with resistance to the outward capillary flow and suppression of coffee ring formation after drying. The impact of decalin on particles is corroborated by actual images acquired from an optical profiler and a scanning electron microscope (SEM). The average particle motion inside the suspension changed by decalin was expressed in terms of mean square displacement (MSD) based on diffusing wave spectroscopy (DWS). Employing multispeckle diffusing wave spectroscopy (MSDWS), the rapid motion or β-relaxation of particles in various suspensions with and without decalin is quantified in early lag time during the drying of droplets. The change in particle dynamics during suspension drop drying, when adding a small secondary fluid, plays a key role in tuning coffee ring patterns.

## 1. Introduction

Among various patterning techniques used for complex colloidal suspensions, inkjet printing is preferred for industrial and academic applications in various fields [1,2]. Micro/nanoscale printing technology uses various polymeric particles or microorganisms as solutes to exert specific functions [3,4,5,6]. It can effectively skip the etching or cutting step, decisively contributing to cost saving in the patterning processes, and also prevents damage to the substrate or sample due to its noncontact and mask-free conditions [7,8]. For these reasons, such an evaporation-driven patterning method provides immense flexibility in the manufacture of various coated patterns. However, the final particulate structure after drying the suspension drops on the substrate exhibits the so-called “coffee ring effect,” which denotes an unintended pattern of nonuniform particle distribution [9].

The coffee ring effect represents a ring-like deposition of particles at the periphery of dried suspension drops [10,11]. The difference in evaporation rate between the center and the edge of a droplet induces an outward capillary flow, resulting in a nonuniform ring-like deposition. Various studies have been explored to utilize and suppress the coffee ring phenomenon [12,13,14,15]. The coffee ring effect can be ingeniously applied via drop coating deposition Raman (DCDR) technique [16] or simple separation process (known as nanochromatography) for biomaterials of various sizes [17]. In addition, the coffee-ring lithography has been developed as a simple one-step fabrication process that aligns particles at the periphery through solvent evaporation [18,19,20].

In general, placing the particles on the drop surface prevents suspension droplets from developing into a coffee ring structure during drying. For example, the self-assembly of particles [21,22], the surface capture effect due to increased temperature [23], the capillary interaction of surfactants [24,25], and altered particle shape [26,27,28] facilitate particle trapping on the surface of a suspension drop, resulting in a uniform particle arrangement.

Specific strategies can be applied to control the radial capillary flow toward the drop periphery to alleviate the coffee ring effect. Polymer additives in the particulate suspension are known to reduce the capillary flow by increasing the suspension viscosity [29]. Large particles or aggregates might also mediate resistance to evaporation-driven flows during drying operations [30,31]. Further, the heterogeneous particles in multimodal suspensions can be a reasonable alternative [32,33] for regulating coffee ring formation.

It is also important to identify the particle mobility in real time, which affects radial capillary flow during suspension drying. Noncontact light scattering technique is one of the best measurements for this purpose. Diffusing wave spectroscopy (DWS) is a practical method used to measure particle motions in terms of mean square displacement (MSD) and rheological properties at high frequencies in dense suspension systems [34,35]. In addition, multispeckle diffusing wave spectroscopy (MSDWS) is utilized to monitor the fast motion of particles in nonergodic particulate suspensions, such as gelation, drying, and curing [36,37,38,39].

In this study, the effects of small amounts of immiscible secondary fluid on particle motions and coffee ring structures in polystyrene (PS) particulate suspensions were investigated, mainly incorporating DWS and MSDWS light scattering techniques. Hydrophobic decalin, which is a hydrocarbon oil, was introduced as the particle connector in water-borne suspensions, inducing permanent and capillary flow-resistant agglomerates of varying size. DWS was used to predict the short-time diffusivity of particles in suspensions. Rapid movement (i.e., β-relaxation dynamics) of PS particles during the drying process, which is directly connected to the dried droplet patterns, was then analyzed in real time using MSDWS. The morphological structures of the final droplets both with and without decalin under different particle concentrations were compared using a scanning electron microscope (SEM) and a three-dimensional (3D) optical profiler.

## 2. Materials and Methods

### 2.1. Preparation of Polystyrene Suspensions

Spherical PS particles of 1 μm in diameter were prepared as described previously [28,33,36]. Hydrophobic decalin was added to suspensions of several particle concentrations by 1 wt% of the total mixture to modify the particle configuration and mobility (Table 1). Based on the inspiration of capillary suspension systems [40,41], the role of a very small amount of decalin (1% by weight here, which does not significantly affect aqueous properties) was identified in water-borne suspension drying. The suspensions were homogeneously mixed with a high-speed homogenizer and treated with an ultrasonic processor (VC-505, Sonics & Materials, Newtown, CT, USA) for 10 min. The weight ratios of decalin to PS were 0.2 for PS-5-D, 0.1 for PS-10-D, and 0.05 for PS-20-D suspensions, as listed in Table 1. Note that this weight ratio would be an important factor on the formation of particles clusters.

### 2.2. Mean Square Displacement Based on Diffusing Wave Spectroscopy Analysis

The effect of hydrocarbon oil on the diffusivity of PS particles in a suspension under equilibrium (or ergodic) state was characterized using DWS (BI-200SM, Brookhaven Instruments, Brookhaven, NY, USA) [34,35]. The prepared PS suspensions were placed inside a 1-μm-thick quartz cell. The cell was placed in the DWS cell holder and illuminated with a He-Ne laser at a wavelength of 637 nm. Multiple scattering inside the cell was expressed using an intensity autocorrelation function (*g*_2_, Equation (1)) based on time-independent fluctuation collected by the photon detector.

(1) $$g_{2}\left(t\right)=\frac{\langle I\left(t_{0}\right)I\left(t_{0}+t\right)\rangle }{{\langle I\left(t_{0}\right)\rangle }^{2}},$$

where *I(t_0_)* and *I(t_0_+t)* represent the light intensities at initial and lag (delay) times, respectively. 〈…〉 denotes the ensemble average [34].

The intensity autocorrelation function (*g*_2_) is then converted to the field autocorrelation function (*g*_1_) using the Siegert relation (Equation (2), [34])

(2) $$g_{2}\left(t\right)=1+\beta {\left|g_{1}\left(t\right)\right|}^{2},$$

Substituting the *g*_1_ from Equation (2) into Equation (3), the mean square displacement (MSD, $\langle \Delta r^{2}\left(t\right)\rangle$) of particles in various suspensions can be determined [34].

(3) $$g_{1}\left(t\right)=\int_{0}^{\infty }P\left(s\right)\exp\left(-\frac{1}{3}k_{0}{}^{2}\langle \Delta r^{2}\left(t\right)\rangle \frac{s}{l^{*}}\right)ds,$$

where *k_0_* is the incident wavevector of light during the scattering. *P*(*s*) denotes the distribution function of the path length *s*, providing information about the history of multiple scattering. Transport mean free path (l*) represents the average distance of light in a random direction.

The MSD indicates the time-dependent fluctuation of particle position, which is strongly related to the diffusive motion of particles inside the suspension. MSD represents a quantitative measure of short-term self-diffusion coefficient (*D*_s_) of particles, as shown below [35].

(4) $$\langle \Delta r^{2}\left(t\right)\rangle =6D_{s}t.$$

### 2.3. β-Relaxation of Particles Using Multispeckle Diffusing Wave Spectroscopy

MSDWS, a modified version of conventional DWS, was employed to understand the β-relaxation dynamics (fast motion) of particles during suspension drying. The volume of the initial suspension droplet was 1 μL. The drying temperature and humidity were maintained at 25 ± 0.5 °C and 50% ± 2%, respectively. Using the line scan CMOS (complementary metal oxide semiconductor) camera (Basler Vision Technologies, Ahrensburg, Germany) the speckle images for 0.1 s lag time were captured every 1 min until the drying was completed. The drying dynamics and relaxation behavior of particle motion within the suspension were expressed in terms of autocorrelation function (*g_2_*-1, Equation (5), [37]) by analyzing the change of the scattering images.

(5) $$g_{2}\left(t_{w},\tau \right)=\frac{\langle I\left(t_{w}\right)I\left(t_{w}+\tau \right)\rangle }{\langle I\left(t_{w}+\tau \right)\rangle \langle I\left(t_{w}\right)\rangle },$$

where *t_w_* and *τ* are the drying time and lag time, respectively. *g_2_*-1 indicates the correlation degree of particles between the initial measuring time and the lag time.

The autocorrelation function of the MSDWS is based on the nonergodic nature of the nonstationary drying process in contrast to the DWS case involving only an ergodic sample. Moreover, when the correlation function decreased to 0.6, the characteristic times for β-relaxation dynamics in the given suspension cases were compared at all drying times. Additional details of the operation and conditions of MSDWS test are described in previous studies [28,33,36,38].

### 2.4. Visualization of Dried Suspension Droplets

The effect of a small amount of secondary fluid on the drying mechanism and coffee ring suppression was carefully analyzed by comparing the particle distributions after complete drying of the suspension droplets. The 3D coffee ring patterns of PS suspension drops were observed using a noncontact optical surface profilometer (NT-1100, Veeco, Plainview, NY, USA) [28] and SEM (SU-70, Hitachi, Tokyo, Japan).

## 3. Results and Discussion

### 3.1. Morphological Changes of Particles Induced by Hydrocarbon Oil Addition

**Figure 1:**
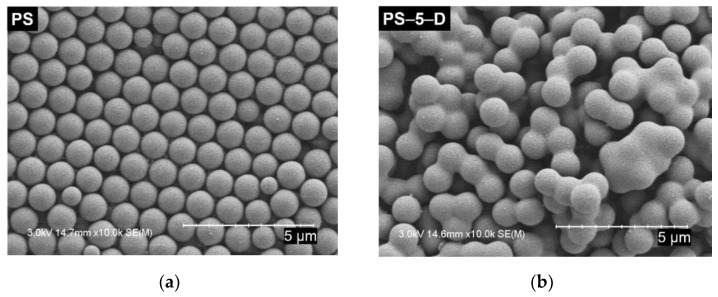

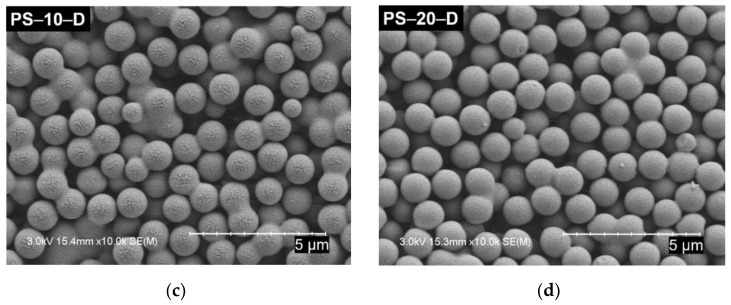
SEM images of PS particles with and without decalin: (**a**) PS without decalin, (**b**) PS-5-D, (**c**) PS-10-D, and (**d**) PS-20-D.

The configuration of PS particles in suspensions with and without a small amount of decalin was inspected using the SEM images, as shown in Figure 1. PS particles (Figure 1a) were spherical in shape with an average diameter of 1.1 μm. When the small amount of decalin was added, random particle clusters were created at all particle concentrations (Figure 1b–d) used in this study. Since the tiny seeds of hydrophobic decalin dispersed through a high-frequency ultrasonic processor act as the linkage point between PS particles, the PS-5-D sample with a high decalin/particle ratio showed the most number of clusters with many linkage points, whereas the PS-20-D showed the least number.

### 3.2. Prediction of Diffusivity of Polystyrene Particles in Various Suspensions

**Figure 2:**
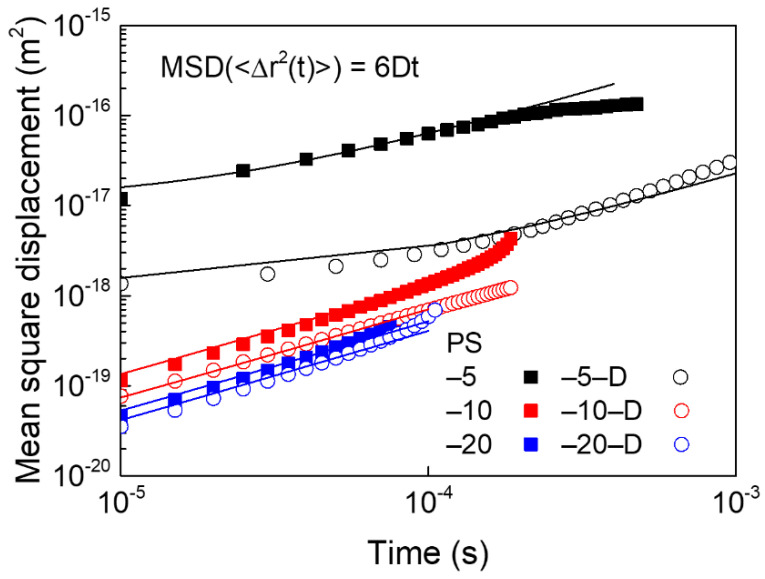
MSD data of PS particles in suspensions treated with and without decalin.

The MSD data for PS particles in suspensions with and without decalin, and the corresponding fitted lines of Equation (4) are displayed in Figure 2 [34,35]. The measurement of diffusive motion can be used to explain the deviation between the current particle position and the initial reference position. In the suspension case without decalin, the MSD data of PS particles were substantially decreased with increasing concentration, indicating that particles had more free space to migrate at lower concentrations. When decalin was added to the suspension, the corresponding MSD was reduced due to the formation of particle clusters in comparison to cases without decalin, especially in PS–5–D with a high decalin/particle ratio.

The short-time diffusion coefficient (*D*_s_) was predicted by fitting the MSD data, which represents the particle motions under short lag time [34,35]. The diffusivities of suspensions without decalin were 8.41 × 10^−15^ (PS-5), 2.18 × 10^−15^ (PS-10), and 1.19 × 10^−15^ (PS-20) cm^2^/s, suggesting reduced mobility at high particle concentrations. It was evident that the increased number of particles in the suspension lowers the space between particles and eventually hinders particle mobility. Addition of hydrocarbon oil led to a decrease in the corresponding diffusivities to 3.64 × 10^−15^ (PS-5-D), 1.17 × 10^−15^ (PS-10-D), and 1.09 × 10^−15^ (PS-20-D) cm^2^/s, respectively. The mobility of PS caused by the addition of decalin into the suspension was remarkably reduced in the case of lower particle concentration case with a high decalin/particle ratio.

### 3.3. Relaxation Dynamics of Particles during Suspension Drop Drying

**Figure 3:**
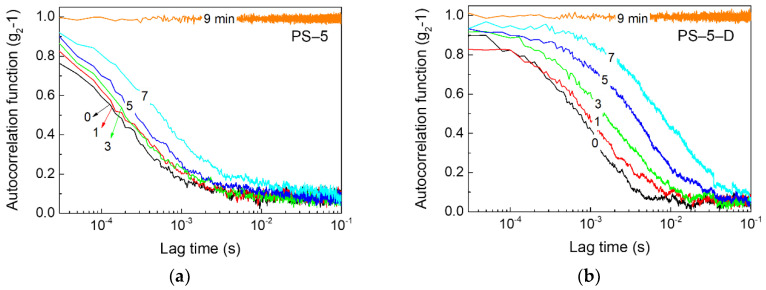

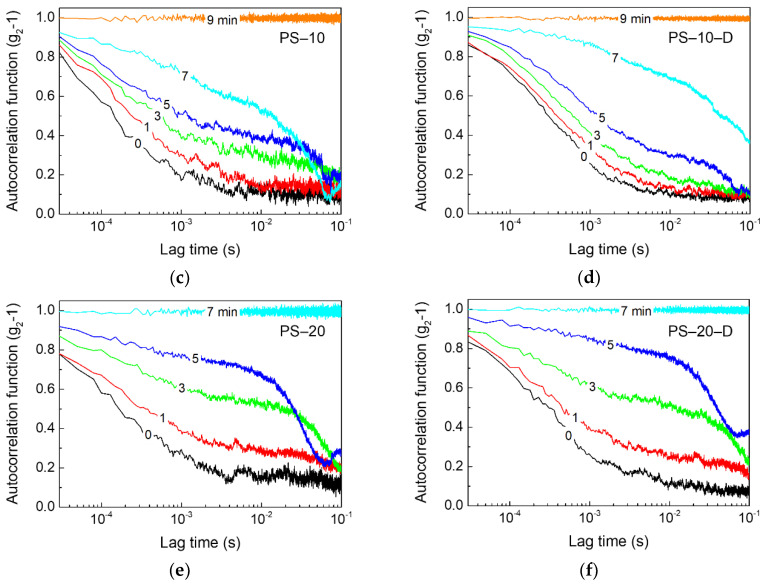
Autocorrelation function curves of PS suspensions with and without decalin during drying. (**a**) PS-5, (**b**) PS-5-D, (**c**) PS-10, (**d**) PS-10-D, (**e**) PS-20, and (**f**) PS-20-D. The number on the data line indicates the drying time.

Rapid Brownian motions of PS particles in a suspension drop were characterized by MSDWS during drying [28,33,36]. The time-dependent autocorrelation functions [*g*_2_-1 in Equation (5)] for suspension systems with and without oil were plotted along the lag time during the entire drying period in Figure 3. In all cases, the autocorrelation function curves increased with drying time, demonstrating that the particles were highly correlated at later drying times. During the early stages of drying, the autocorrelation functions decreased rapidly at short lag time regimes showing fast Brownian motion, and the correlation between particles inside the suspension vanished. As the drying continued, particle motion was severely restricted due to solvent evaporation, and thus the correlation gradually increased. After the drying process was terminated, the autocorrelation function was constant at a value of 1. Based on the comparison of suspension drops with and without decalin, the particles were found to be strongly correlated along the lag time in decalin-added suspensions.

**Figure 4:**
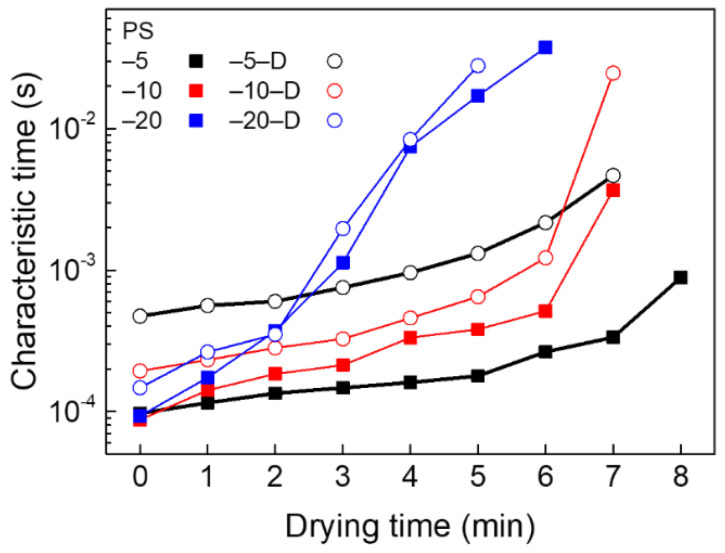
Characteristic times for β-relaxation behaviors of given PS suspensions during drying.

A comprehensive analysis of the drying features of various suspensions based on the characteristic times of β-relaxation modes is presented in Figure 4, which presents the lag times at an autocorrelation function value of 0.6 [28,33,36]. In suspensions without decalin, the characteristic time of PS-20 increased dramatically during evaporation, signifying an abrupt reduction in PS mobility inside a suspension drop due to dense packing [36]. However, the PS-5 case showed a rather slow change in characteristic time, because the particle motion was not relatively restricted, compared with other cases. The characteristic times exhibited different levels when a small amount of decalin was added into the suspensions. The apparent particle motion inside the decalin-containing suspensions was decreased compared to the suspensions without decalin (i.e., increasing characteristics times), which was obvious from the behavior of particle clusters. The characteristic times with the addition of decalin were greatly increased for initially low concentrations of suspension drops. Overall, it is clear that addition of a small amount of hydrocarbon oil suppresses the rapid motion of suspensions by creating particle clusters.

### 3.4. Coffee Ring Patterns of Dried Suspension Droplets

**Figure 5:**
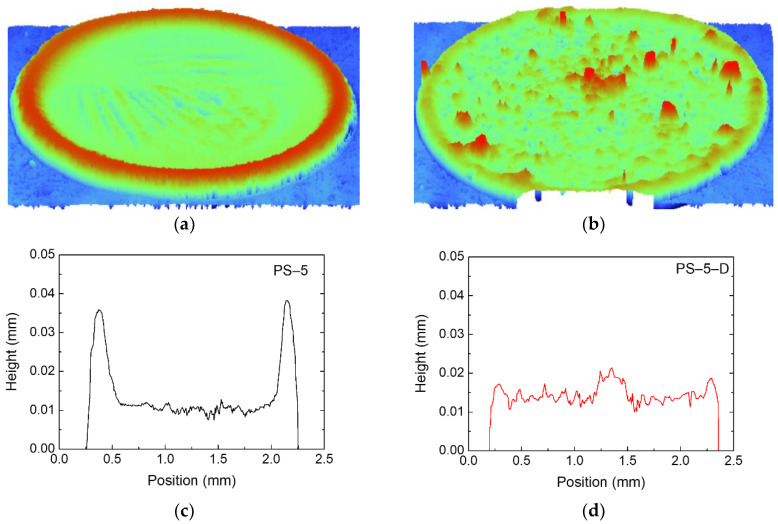
Three-dimensional images of dried (**a**) PS-5 and (**b**) PS-5-D samples, and the corresponding heights of (**c**) PS-5 and (**d**) PS-5-D samples.

The final dried patterns of the PS-5 and the PS-5-D suspensions were compared using a 3D optical profiler. PS–5 (Figure 5a) shows a clear coffee ring structure with thick particle layers on the droplet edge. In the PS-5-D case (Figure 5b) treated with a small amount of decalin, a more uniform but slightly bumpy structure developed after drying. The two-dimensional (2D) heights of dried droplets in Figure 5c,d also reveal the reduced coffee ring effect in the PS-5-D case compared with the PS-5 case, because particle clusters generated by decalin in the PS-5-D delayed the capillary flow of particles toward the drop periphery.

**Figure 6:**
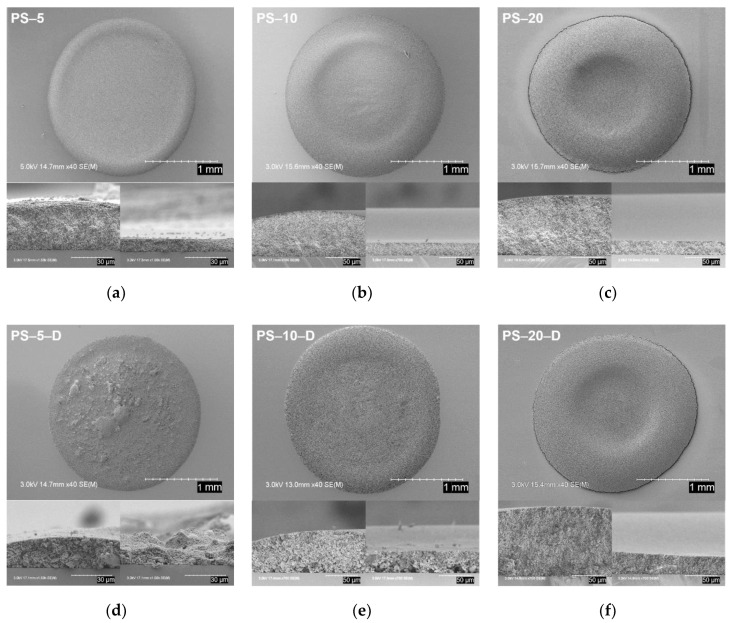
Final SEM images based on the top and cross-sectional views of dried suspension droplets: (**a**) PS-5, (**b**) PS-10, (**c**) PS-20, (**d**) PS-5-D, (**e**) PS-10-D, and (**f**) PS-20-D.

The particle deposition patterns and cross-sectional images of the entire suspension droplets after complete drying were obtained via SEM. The coffee ring structures are clearly visible from the top of dried suspension droplets without decalin (Figure 6a–c). The ring size increased in width as the particle concentration increased. In addition, the edge of the dried structure was higher than the center because the dominant outward capillary flow in the droplet caused repositioning of more particles around the periphery of the drop [42]. However, suspension droplets with oil displayed various patterns of particle deposition. In Figure 6d, the difference in height at the edge and the center of the dried droplet of the PS-5-D showed less variation, but the clusters inside the droplet were easily observed. The coffee ring of the PS-10-D was apparently less remarkable than that of the PS-10 due to the capillary flow suppression caused by particle clusters [43,44]. However, the dried pattern of the PS-20-D appears to be very similar to the PS-20 because the relatively low decalin/particle ratio did not effectively modify the suspension properties to alter the coffee ring effect. Accordingly, a uniform particulate structure after evaporation of suspension drops can be obtained by optimizing the ratio between the secondary fluid and the particles.

## 4. Conclusions

Drying dynamics and coffee ring patterns of PS suspension drops were carefully tuned by the addition of a small amount of secondary fluid (decalin), as a particle connector. Particle motion in stationary and nonstationary evaporation states was interpreted via light scattering methods such as DWS and MSDWS, respectively. The manipulation to multimodal systems of polymeric PS particles fused by decalin, whose size was dependent on the particle concentration, was captured by the magnified SEM images. Particle motions in various suspensions with and without decalin under equilibrium condition before drying were quantified from MSD data in DWS tests. A small amount of decalin significantly reduced the average particle motion at low initial concentrations, but the effect was diminished with increasing particle content, depending on the cluster formation. Based on MSDWS results obtained during the drying of suspension drops, the particles inside decalin-added suspensions were strongly correlated with each other, compared with decalin-free suspension at similar drying times. This finding substantiates that the particle clusters formed in decalin-treated suspensions obviously slowed the outward capillary flow. Real-time particle motion analyzed by light scattering techniques during drying is closely related to the final particle configurations and the coffee ring patterns after complete drying. The particle distributions in decalin-added suspensions after drying tended to reduce the coffee ring formation by appropriately controlling the ratio between the particles and decalin.

## Figures and Tables

**Table 1:** Sample information of PS particulate suspension treated with and without decalin.

Sample	Particle (P)*	Decalin (D)*	D/P in Weight
PS-5	0.05 g	–	–
PS-10	0.10 g	–	–
PS-20	0.20 g	–	–
PS-5-D	0.05 g	0.01 g	0.2
PS-10-D	0.10 g	0.01 g	0.1
PS-20-D	0.20 g	0.01 g	0.05

* Based on the total 1 g weight of suspension.
